# Interdependent Regulation of Alternative Splicing by Serine/Arginine-Rich and Heterogeneous Nuclear Ribonucleoprotein Splicing Factors

**DOI:** 10.3390/genes17010078

**Published:** 2026-01-09

**Authors:** Megan E. Holmes, Klemens J. Hertel

**Affiliations:** Department of Microbiology and Molecular Genetics, University of California Irvine, Irvine, CA 92697, USA

**Keywords:** alternative splicing, SR proteins, hnRNPs, exon inclusion

## Abstract

Background: Alternative pre-mRNA splicing is a combinatorial process involving serine/arginine-rich (SR) and heterogeneous nuclear ribonucleoprotein (hnRNP) splicing factors. These proteins can silence or enhance splicing based on their expression levels and binding positions. Objectives: To better understand the combinatorial and interdependent regulation between SR and hnRNP splicing factors during alternative splicing. Methods: Computational analyses were performed using cell knockdown and binding datasets from available databases. Results: Analyses of differential splicing data for 9 SR proteins and 21 hnRNP knockdowns revealed statistically significant interdependent regulation among several RNA-binding protein (RBP) combinations, albeit at different levels. Neither SR proteins nor hnRNPs showed strong preferences for collaborating with specific RBP classes in mediating exon inclusion. While SRSF3, hnRNPK, hnRNPC, and hnRNPL stand out as major influencers of alternative splicing, they do so predominantly independent of other RBPs. Minor influencers of alternative splicing, such as hnRNPDL and hnRNPR, predominantly regulate exon inclusion in concert with other RBPs, indicating that exon inclusion can be mediated by both single and multiple RBPs. Interestingly, the higher the number of RBPs that regulate the inclusion of an exon, the more variable exon inclusion preferences become. Interdependently regulated exons are more modular and can be characterized by weaker splice sites compared to their independently regulated counterparts. A comparison of RBP interdependence between HeLa and other cell lines provides a framework that explains cell-type-specific alternative splicing. Conclusions: Our study highlights the importance of the interdependent regulation of alternative exons and identifies characteristics of interdependently regulated exons that differ from independently regulated exons.

## 1. Introduction

During splicing, introns are excised from the pre-mRNA strand, and the remaining exons are ligated to form an mRNA strand [1,2,3]. Alternative splicing (AS) events are instances where exons are skipped (exon skipping), introns are retained (intron retention), or alternative splice sites are chosen (alternative 3′/5′ selection) [2,4,5]. Given the limited number of genes in eukaryotic genomes, AS is essential for diversifying the eukaryotic proteome [6,7]. AS is responsible for a large percentage of that diversity, with the majority of human multi-exon genes having AS events [8,9,10].

Exon skipping (ES) is the most frequent form of AS in which changes in exon inclusion generate different mRNA isoforms. As with all forms of AS, ES is a combinatorial process that is regulated by both cis-acting elements and trans-acting factors [11,12]. Two classes of trans-acting factors, the SR protein and hnRNP families, have been shown to affect spliceosomal assembly through similar mechanisms [1,2]. These splicing factors are known to be involved in numerous disease pathologies, including cancers and neurological disorders though the regulation of AS events [13]. We hypothesized that it is likely that the SR and hnRNP trans-acting factors interdependently regulate ES. Here, interdependent regulation is defined as regulation through direct and/or indirect effects. Direct effects consider instances where the interaction of both RNA-binding proteins (RBPs) with the pre-mRNA affects exon inclusion. Indirect effects may occur when the altered expression of an RBP influences the expression or splicing pattern of another RBP, which could potentially influence exon inclusion. Gaining insight into the interdependent regulation of these splicing factors may provide new understandings of disease mechanisms by resolving different pathway targets and highlighting roles of less studied SR and hnRNP splicing factors in disease. To investigate the interdependent regulation of ES by these two protein families in humans, we turned to complementing large-scale RBP knockdown datasets, which contain RNA-seq knockdown data for over 300 RBPs in Hela, HepG2, and K562 cell lines [14,15]. Here, we utilize the data for all SR and hnRNP knockdowns available in the RBP knockdown datasets to determine the prevalence of interdependent regulation among members of the SR and hnRNP families of splicing regulators.

This multidimensional analysis of interdependence shows that 70% of the 435 possible pairs of evaluated SR and hnRNPs interdependently regulate exon inclusion at varying degrees. Exon inclusion can result from the action of one or several RBPs. Notably, as the number of RBPs governing exon inclusion increases, so does the variability in exon inclusion preferences. Exons regulated interdependently exhibit weaker 5′ splice sites when compared to those regulated independently. These results underscore the significance of interdependent regulation in alternative exon inclusion and pinpoint unique characteristics of such exons that are distinct from independently regulated ones.

## 2. Materials and Methods

### 2.1. Identification of Alternative Splicing Changes Using Publicly Available Datasets

Our analysis of interdependent regulation was carried out on human 30 pre-mRNA splicing factors consisting of 9 SR proteins and 21 hnRNPs, using data from readily available datasets in HeLa cells [14] or HepG2 and K562 cells [15]. To find the total number of exons affected by the knockdown of each splicing factor, we used the differential splicing analysis files from the dataset. Each file was filtered for exons that experience greater than a 15% change in inclusion levels upon knockdown of the respective RBP, analogous to the original analysis [14]. The remaining exons were determined to have been affected by the knockdown of that RBP.

Analysis of enhancing and silencing activity was performed by binning the affected exons of each RBP based on whether they had a positive inclusion level difference (increase in inclusion) or a negative inclusion level difference (decrease in inclusion) upon RBP knockdown. Exons with a decrease in inclusion level are assumed to experience an enhancing effect from the RBP in wildtype conditions, while exons with an increase in inclusion level are assumed to experience a silencing effect from the RBP in wildtype conditions.

### 2.2. Identifying RBP Pairs That Exhibit Statistically Significant Interdependent Regulation

To identify statistically significant interdependent regulation between each of the 435 possible non-duplicate RBP pairs, we used the survival function:

sfm:N,M,n=1−∑i=0m−1Mi·N−Mn−iNn

- m: the number of exons present in both filtered differential splicing analysis files for a given pair.
- N: the total number of exons in the unfiltered differential splicing analysis files combined for the proteins of interest
- M: the total number of exons in the filtered differential splicing analysis file for the first RBP of interest
- n: the total number of exons in the filtered differential splicing analysis file for the second RBP of interest

Exon skipping differential splicing analysis files comparing wildtype HeLa and the HeLa SR/hnRNP knockdowns were filtered for exons with an inclusion level difference >15% to create the ‘filtered’ differential splicing analysis files. ‘Unfiltered’ differential splicing analysis files refer to the raw files without duplicates. Pairs whose survival function returned a *p*-value ≤ 0.05 exhibit statistically significant interdependent regulation.

### 2.3. Clustering of RBP Interdependence Activity

Clustering of the interdependence matrix (Supplemental Figure S2) was carried out using the R package pheatmap 1.0.13 [16]. Within pheatmap 1.0.13, hierarchical clustering was performed using Euclidean distance and complete linkage (as implemented in the base R function hclust).

### 2.4. Identifying Evidence of Indirect Versus Direct Effects of Interdependence

To identify possible indirect effects of RBP knockdowns, the HeLa cell RBP knockdown dataset was evaluated for instances where the knockdown of an RBP induces changes in the splicing pattern of other RBPs. Differential splicing analyses were used to determine whether the knockdown of an RBP resulted in differential splicing of other SR/hnRNPs. Significant splicing pattern changes in SR/hnRNPs resulting from the knockdown of another SR/hnRNP were characterized as instances where an RBP had at least one exon that exhibited a >15% difference in inclusion.

### 2.5. Correlating Exon Features with Exons Affected by Varying Numbers of RBP Knockdowns

Alternatively spliced exons were binned by the number of RBPs affecting their inclusion. Exon parameters such as exon length, the upstream and downstream intron lengths, the 3′ and 5′ splice site strengths, and sequence conservation (PhyloP score) were extracted from UCSC genome browser tracks and from MaxEnt splice site score calculators [17,18]. The averages of these characteristics were then compared across each exon bin. A linear regression model was used to determine if any of the characteristics exhibited a linear correlation across groups with increasing numbers of affecting RBPs.

### 2.6. Analysis of Cell Type Specificity

An analysis of cell type specificity was performed using the batch-corrected ENCODE data for all SR/hnRNP knockdowns in HepG2 and K562 cell lines [15]. Using the same method used for the HeLa dataset, the number of RBP pairs that exhibit statistically significant interdependent regulation for both cell lines was identified. Because statistical information was available for the ENCODE datasets, the ‘filtered’ differential splicing analysis files were created by filtering the exon skipping differential splicing analysis files for exons with an FDR ≤ 0.05 and an inclusion level difference >10%. The relative importance of splicing factors in different cell lines was calculated for each of the RBPs with available knockdown data in HeLa, HepG2, and K562 cell lines. The relative level of importance was calculated by normalizing the total number of affected exons for each RBP in each cell line by the largest number of exons affected by any RBP in each cell line.

### 2.7. Data Sources

The alternative splicing data for splicing factor knockdowns in HeLa cells that were used in this analysis are available in Supplemental Table S1 [14]. The alternative splicing data for splicing factor knockdowns in HepG2 and K562 cells are available at the ENCODE Data Coordination Center (https://www.encodeproject.org/) [15]. Knockdown efficiency information is available for the ENCODE dataset (HepG2 and K562 cells), with all RBP knockdowns meeting a minimal 50% knockdown efficiency requirement, as measured by PCR and Western analysis [15]. Dataset information and file links for the knockdown datasets used are listed in Supplemental Table S1.

## 3. Results

### 3.1. The Influence of Individual Splicing Regulators on Exon Inclusion

**Figure 1:**
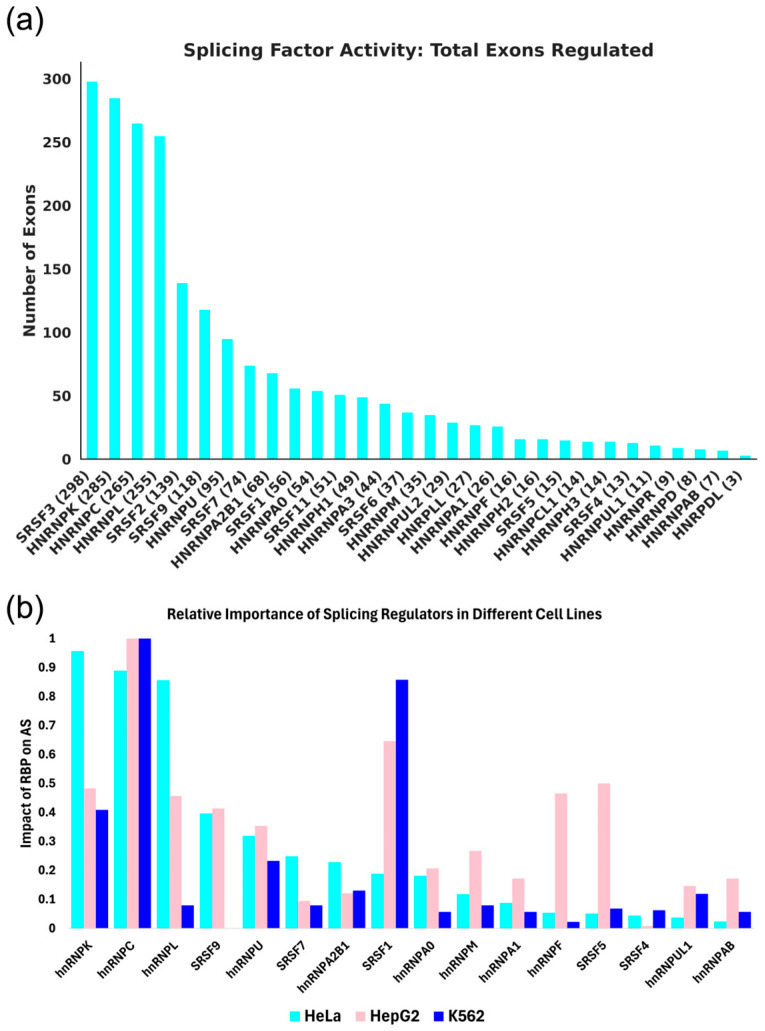
The influence of individual splicing regulators on exon inclusion. (**a**) Bar plot showing the total number of exons with more than a 15% change in inclusion level upon RBP knockdown in HeLa cells. (**b**) Grouped bar plot comparing the normalized impact of each RBP knockdown shared between the HeLa, HepG2, and K562 knockdown datasets. The impact of an RBP on AS was generated by normalizing the number of exons affected by an RBP to the largest number of exons regulated by any RBP within each of the cell lines evaluated.

SR and hnRNP splicing regulators have been shown to regulate exon inclusion by modulating the recruitment of spliceosomal components to splice sites [9,19,20,21,22]. To understand the extent to which RBPs influence exon recognition, we evaluated how many exon inclusion events were significantly affected by the knockdown of 30 individual splicing regulators in HeLa cells [14]. For this SR and hnRNP analysis, we took advantage of a recently published dataset that reports on alternative splicing differences for more than 300 RNA-binding proteins [14]. The broadness of an SR or hnRNP splicing regulator’s role in exon inclusion varies greatly between the analyzed splicing regulators (Figure 1a, Supplemental Table S2). For example, 298 exons were identified with significant changes in inclusion upon the knockdown of SRSF3, while only 3 exons were identified with changes in inclusion upon the knockdown of hnRNPDL.

An analogous analysis was performed using batch-corrected SR and hnRNP knockdown ENCODE datasets [15] available for HepG2 and K562 cells (Supplemental Figure S1, Supplemental Tables S3 and S4). This analysis resulted in the identification of fewer significantly affected alternative exon inclusion events, most likely a consequence of the stringent batch correction. A comparison between the datasets analyzed demonstrates that the role of an SR or hnRNP splicing regulator, when normalized to the total number of affected exons, can vary between cell lines. While the knockdown of some splicing regulators, such as hnRNP C or U, elicited a comparable relative number of significant splicing changes in all cell lines tested, the knockdown of other splicing regulators, such as hnRNPL and SRSF1, displayed much more varied levels of alternative splicing outcomes (Figure 1b). We conclude that the extent to which an RBP influences exon inclusion levels varies across the different RBPs and the cell lines analyzed.

### 3.2. SR and hnRNP Splicing Factors Interdependently Regulate Exon Inclusion

**Figure 2:**
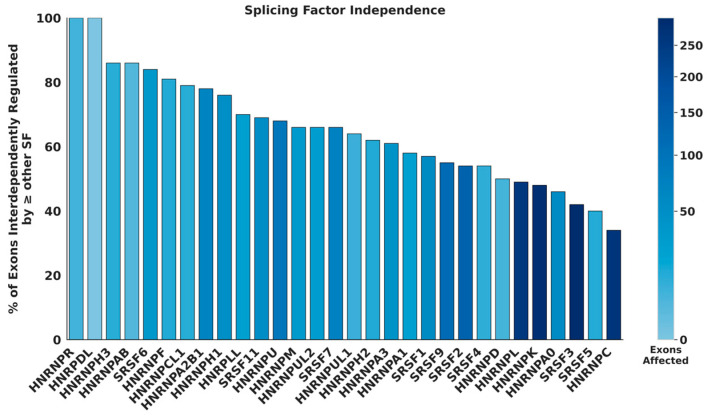
SR and hnRNP splicing factors interdependently regulate exon inclusion. Bar plot showing the percentage of each RBP’s total affected exons in HeLa cells that are also affected by another RBP. The intensity of the bar color reflects the frequency of AS upon RBP knockdown.

**Figure 3:**
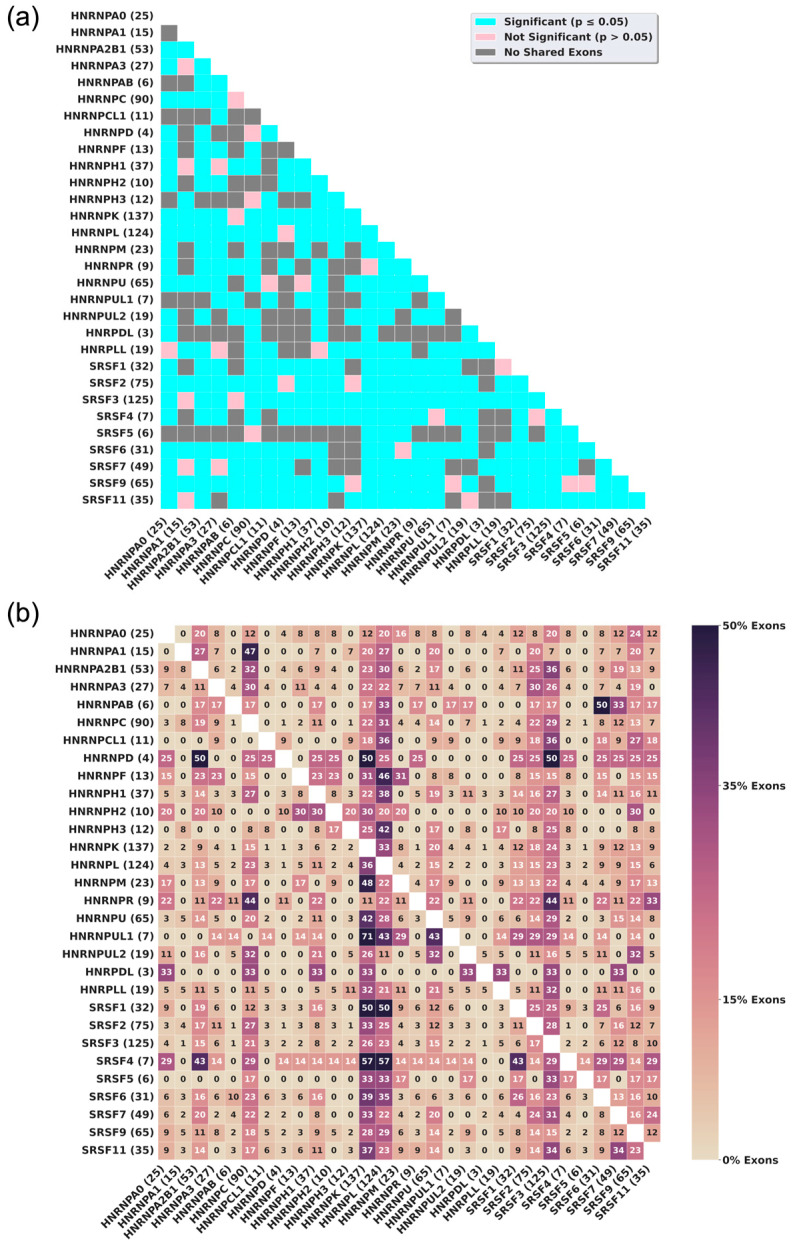
SR and hnRNP splicing factors interdependently regulate exon inclusion at statistically significant levels. (**a**) Matrix indicating which of the pairs of the 30 splicing factors analyzed show statistically significant interdependent regulation in HeLa cells. The number in parentheses after each RBP name is the total number of exons with AS that are also affected by another RBP. Cyan indicates statistically significant pairs (*p* ≤ 0.05), pink indicates statistically insignificant pairs (*p* > 0.05), and gray indicates no overlap between pairs. (**b**) Heatmap displaying the percentage of alternatively spliced exons that are independently regulated by another RBP in HeLa cells. The numbers in parentheses are the total number of exons interdependently regulated by that RBP. The values in the cells are the percentages of the interdependently regulated exons from the RBP in each row that are also regulated by the RBP defined by the columns.

Using the alternative splicing information, we evaluated which of the 435 pairs of SR and hnRNP splicing factors available for HeLa cells exhibit statistically significant interdependent regulation of exon inclusion. Here, interdependent regulation is defined as the direct or indirect regulation of an exon by two or more RBPs. For the majority of the RBPs analyzed, interdependently regulated exons make up at least half of the affected exon population, indicating a strong likelihood that some of the pairs exhibit statistically significant interdependent regulation (Figure 2). The statistical analysis of interdependence between the pairs shows that 70% of the possible splicing regulator pairs exhibit interdependent regulation (Figure 3a). These observations indicate that most SR and hnRNPs interdependently regulate exon inclusion in HeLa cells. A splicing factor’s level of interdependent splicing regulation varies. This is demonstrated by the difference in the percentage of exons that are also affected by other splicing factors (Figure 3b). For example, hnRNPK-affected exons have low interdependent regulation percentages, while SRSF4 has higher interdependent regulation percentages (Figure 3b, rows). However, hnRNPK displays interdependent regulation of splicing with many other splicing regulators, while SRSF4 is more selective for a subset of hnRNP proteins in its ability to mediate interdependent exon inclusion (Figure 3b, columns). The data further suggest that the exons affected by hnRNPR are interdependently regulated by more than one other RBP. By contrast, hnRNPC has a larger proportion of exons that are not interdependently regulated (Figure 2 and Figure 3b), suggesting that hnRNPC modulates exon inclusion mainly independent of other splicing regulators. Interdependent regulation occurs at varying levels between different pairs of splicing regulators without a clear trend of RBP class preferences. This observation is supported by cluster analyses (Supplemental Figure S2). RBPs affecting fewer exons appear to exhibit greater levels of interdependent splicing regulation. A lower percentage of pairs exhibiting interdependent regulation was observed in HepG2 and K562 cells. However, this analysis is limited by the much lower number of significant alternative splicing events detected in batch-corrected HepG2 and K562 datasets (Supplemental Figure S3a,b).

### 3.3. Enhancing and Silencing Activity of SR and hnRNPs Varies Based on Interdependence

**Figure 4:**
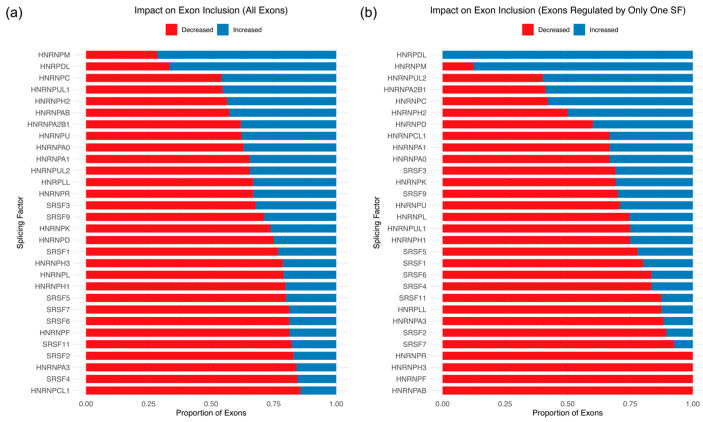
Enhancing and silencing the activity of SR and hnRNP proteins varies based on interdependence. (**a**) Stacked bar graph showing the percentage of exons with decreasing (red) and increasing (blue) inclusion levels upon knockdown of each RBP in HeLa cells. (**b**) The same as (**a**), but for just the independently regulated population of exons (exons only affected by one RBP).

SR proteins are primarily known to act as splicing enhancers, while hnRNP proteins are best known to silence splicing [1,2,23,24]. However, it is also appreciated that splicing regulators can act as both splicing silencers and enhancers, presumably dictated by their binding relative to regulated splice sites [15,25,26,27,28,29,30]. To determine whether SR and hnRNP proteins preferentially act as silencers or enhancers, we computed the percentage of exons displaying an increase or decrease in inclusion level upon RBP knockdown (Figure 4). This analysis was carried out for two different populations of alternatively spliced exons: the total population consisting of every exon affected by the knockdown of the RBP of interest, and the independent population consisting of all exons that are only affected by the RBP of interest. In agreement with previous results, all splicing regulators exhibit both silencing and enhancing activity on the total population, albeit with different preferences [28,30]. For example, knockdowns of most SR splicing regulators resulted in preferential exon inclusion loss, supporting the notion that SR proteins primarily act as splicing enhancers (Figure 4a). The inverse is observed for several hnRNP splicing regulators, consistent with their general role as splicing silencers (Figure 4b). The splicing tendencies of SR proteins shifted to increased silencing when exon inclusion is mediated by at least one other RBP (interdependent population). The splicing-enhancing function of SR proteins is amplified if it is the only RBP acting on exon inclusion (independent population) (Figure 4a). The hnRNPs analyzed exhibited both preferential silencing and activating activity, especially within the independent exon population. With minor deviations, these enhancing and silencing preferences are also observed for the RBP knockdown analysis in HepG2 and K562 cells (Supplemental Figure S4). We conclude that SR and hnRNP splicing regulators differentially affect exon inclusion levels, and their splicing/enhancer tendencies are modulated depending on whether or not the exon is interdependently regulated.

### 3.4. Independently and Interdependently Regulated Exon Populations Have Different Characteristics

**Figure 5:**
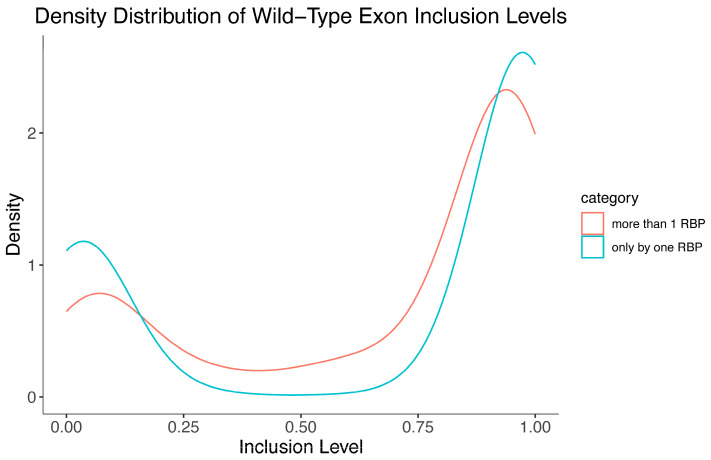
Independently and interdependently regulated exon populations have different wild-type inclusion level distributions. Density distribution of wildtype exon inclusion levels for the independently regulated exon population (affected by only one RBP) and the interdependently regulated exon population (affected by more than one RBP).

Typically, exons are highly or lowly included, with few having intermediate inclusion levels (~50%) [31]. Considering the difference in the enhancing/silencing activity of the interdependent and independent RBP populations (Figure 4), we evaluated whether the wild-type exon inclusion levels of RBP-affected exons differ between the two populations. Interestingly, while the average inclusion levels of both groups were similar, the distribution of each group was observed to be considerably different. The independent population exhibited a stronger bimodal distribution of lowly and highly included exons [31], while the interdependent group exhibited an increase in exons represented by intermediate inclusion levels (Figure 5).

**Figure 6:**
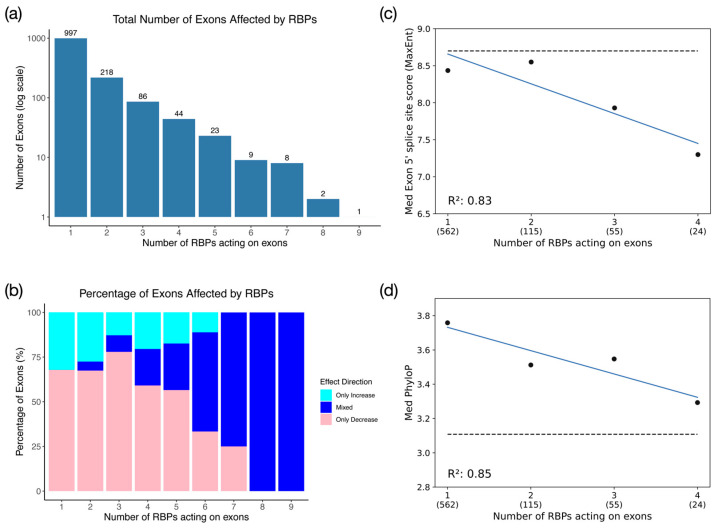
Exon feature bias for exons affected by multiple RBPs. (**a**) Bar graph showing the number of exons that are affected by the knockdown of one or multiple RBPs in HeLa cells. (**b**) Stacked histogram showing how the number of RBPs affecting individual exons dictates preferential increase (teal), decrease (pink), or variable (dark blue) exon inclusion. (**c**) Correlation between exon 5′ splice site score and the number of RBPs affecting individual exons. The dashed line indicates the median exon 5′ splice site score for all internal exons in the human transcriptome. (**d**) The same as (**c**) but for the PhyloP score.

To further break down the differences between the independent and interdependent populations, we determined how many RBPs act on each exon by combining the exon inclusion data for all knockdown datasets and computing how many RBPs can act on each exon. Greater than 70% of alternatively spliced exons are only affected by one RBP (independently regulated). Astonishingly, up to 9 RBPs can affect the inclusion level of a single exon, showing that some exons experience interdependent regulation with multiple RBPs (Figure 6a). Within these populations, we determined the percentage of exons with an exclusive increase or decrease in inclusion level, or the percentage of exons with inclusion levels in either direction (Figure 6b). The data show a preference for RBP-mediated enhancement of exon inclusion. As the number of RBPs affecting an exon’s inclusion level increases, the fraction of the population that mediates both increased and decreased exon inclusion becomes increasingly dominant (Figure 6b). Thus, the cooperation of multiple RBPs in mediating exon inclusion reduces the preferential enhancement of exon inclusion that is typically induced by the actions of a single RBP.

To determine whether exons that are regulated by a different number of RBPs display unique cis-regulatory features, we compared exon length, intron length, splice site scores, and sequence conservation across the various exon populations. The most significant dependencies we observed were negative correlations between the number of RBPs affecting exon inclusion and 5′ splice site scores and sequence conservation scores (PhyloP) (Figure 6c,d). Exons with splice site scores closest to the genome-wide average of splice site scores are preferentially regulated by a single RBP and as the number of RBPs modulating exon inclusion increases, the 5′ splice site strength decreases. Weaker correlations were also found between the number of RBPs affecting exon inclusion and the upstream intron length or exon size (Supplemental Figure S5). In general, splice site scores are lower than the genome average across all populations, suggesting that all of the SR and hnRNP-affected exons have weaker splice site scores than the genome-wide population. Interestingly, the inclusion of exons with weaker splice sites requires a higher number of interdependent RBPs (Figure 6c). Compared to the genome-wide average, the sequence conservation of all populations is elevated. These observations suggest that exons targeted by multiple splicing regulators have evolved to establish a sub-optimal exon recognition potential. Other features, such as 3′ splice site score and downstream intron length, showed insignificant levels of linear correlation.

## 4. Discussion

In this study, interdependence was defined by the overlap of exons between the differential splicing analysis of two RBP knockdowns. The hypothesis that SR/hnRNPs interdependently regulate exon inclusion is supported by this study, as 70% of the 435 possible non-duplicate pairs of the SR/hnRNPs available for the HeLa cell dataset exhibit statistically significant interdependent regulation. A comparable extent of interdependent regulation was observed in HepG2 and K562 cells (ENCODE), indicating that interdependent regulation is not unique to HeLa cells. Many splicing factors interdependently regulate with multiple other splicing factors, with the identities of their partners varying between cell lines (Supplemental Table S5). The broad range in the number of exons that an RBP regulates and the variation in those counts per RBP between cell lines suggest that each cell line has common and unique RBP regulatory influences (Figure 1b). hnRNPK and hnRNPC emerged as two of the most influential splicing regulators, as their knockdown resulted in differential exon inclusion for many exons in all cell lines (Figure 1b). The exons affected by lowly active RBPs have higher levels of interdependent regulation, suggesting that lowly active RBPs may only function in those cell lines as interdependent regulators. A surprising observation was the fact that >70% of exons undergoing alternative splicing upon RBP knockdown appear independently regulated, meaning their inclusion level was only affected by the knockdown of one SR or hnRNP protein (Figure 6a). While other splicing regulators not evaluated in this study could affect such independently regulated exons, a picture emerges that many alternatively spliced exons are often regulated by the activity of only one splicing regulator. A case can be made that the involvement of multiple RBPs in regulating alternative exon inclusion allows the fine-tuning of exon inclusion levels and increased modularity in mediating increased or decreased exon inclusion. If such interdependently regulated exons are associated with disease, the splicing factors involved in controlling exon inclusion could be considered as potential targets for splice correction.

### 4.1. Pairs of RBPs, Family of RBPs, and Trends in Interdependent Regulation

The interdependence matrix (Figure 3b) illustrates what frequency exons affected by the knockdown of an RBP defined on the Y-axis are also affected by the knockdown of RBPs defined by the X-axis. Crucial in evaluating the information presented is considering the total number of interdependently regulated exons that are associated with the knockdown of an RBP. For example, the knockdown of hnRNPR changes the inclusion of 9 exons (Figure 1a), all of which (100%) (Figure 3b) are also regulated by other RBPs. The matrix shows that 44% of hnRNPR’s interdependently regulated exons (4 exons) are also regulated by hnRNPC. Thus, hnRNPC and hnRNPR frequently co-regulate exon inclusion. On the other hand, only 3 of hnRNPK’s 137 affected exons (2%) are interdependently affected by hnRNPA0, suggesting that hnRNPK and hnRNPA0 do not operate frequently on the same set of exons. The clustered matrix also demonstrates that the degree of interdependent regulation between RBPs is not defined by family traits nor is it influenced by whether an RBP is a preferential enhancer or repressor of exon inclusion (Supplemental Figure S2). Thus, the SR and hnRNP splicing regulators display a flexible potential to contribute to exon inclusion.

### 4.2. Direct Versus Indirect Effects Mediating Interdependent Splicing Regulation

Splicing regulators are known to bind the pre-mRNA and mediate splicing activity by influencing the recruitment efficiency of spliceosomal components. Thus, when alternative splicing is detected upon knockdown of a splicing regulator, it is assumed that the difference in an exon’s inclusion level is caused by the loss of the direct interaction between the pre-mRNA and the splicing regulator. While this ‘direct effect’ is logical, it is also possible that indirect effects, such as induced changes in the expression of other splicing regulators, could trigger alternative splicing. The available data (splicing, ENCODE eCLIP binding) provided only limited insights into addressing to what degree the observed splicing changes upon RBP knockdown were caused by direct (loss of RBP binding) or potential indirect effects (altered expression or splicing of other RBPs). No overlap was found when we looked for RBP eCLIP peaks on the alternatively spliced exons affected by the RBP. This result does not disprove direct effects, but it is more likely caused by the stringent filtering of the high-confidence eCLIP peak ENCODE dataset and the stringent batch correction of the ENCODE batch-corrected knockdown datasets [15]. Even if overlap was observed, a correlation between the presence of eCLIP binding peak within and around alternatively spliced exons does not prove an RBP’s involvement in regulating exon inclusion, as the RBPs may be bound to exons before or after splicing occurs. Furthermore, the absence of eCLIP binding does not disprove direct binding, especially when RBP binding occurs within short-lived introns. Nevertheless, direct effects are still highly likely to mediate observed alternative splicing, particularly in the case of exons affected only by a single RBP (independently regulated exons). Alternative splicing analyses demonstrated that the knockdown of an RBP can cause, in a limited number of cases, changes in the splicing pattern of another RBP, raising the potential that indirect effects impact alternative splicing outcomes of RBP knockdowns (Supplemental Figure S6). Interestingly, many of these induced changes in alternative splicing affect the isoform expression of the target RBP, suggesting autoregulation as previously described for several splicing regulators [32]. While it is unknown to what degree these changes in other RBP isoform representation affect alternative splicing, they must be considered as potentially influential when evaluating the alternative splicing landscape of single RBP knockdowns. Thus, alternative splicing changes observed upon RBP knockdown are caused by both direct and indirect effects.

While the analysis of extensive knockdown datasets provided insights into the interdependent regulation of AS, it is unclear if off-target effects could have influenced the results obtained. Both the HeLa and ENCODE datasets were tested and, if necessary, corrected for batch effects, and made use of siRNA pools, scrambled non-target controls, low concentrations of siRNAs, or optimized alignment specificities to reduce off-target effects [14,15]. Given these precautions, off-target effects in the datasets used are minimized; however, they cannot be excluded from potentially influencing the interdependency analysis.

### 4.3. Features of Interdependently Regulated Exons

The extreme bimodal distribution of exon inclusion levels for the independently regulated exons is similar to the exon inclusion distribution of human exons, with an underrepresentation of intermediate exon inclusion levels [31]. Interestingly, the exon inclusion distribution of interdependently regulated exons is noticeably different as intermediate inclusion level exons are increased (Figure 5). These exons are more easily shifted towards inclusion or exclusion than independently regulated exons. This increased modularity likely makes it easier for the balance of the exon’s inclusion to be shifted in either direction by the combined regulation of RBPs. As expected, based on steric hindrance, shorter exons are associated with a lower number of RBPs regulating their inclusion (Supplemental Figure S5), perhaps limited by exon definition parameters.

### 4.4. Cell Type Specificity

By including the batch corrected ENCODE datasets for RBP knockdowns in HepG2 and K562 cells, we were able to compare interdependent regulation in HepG2, K562, and HeLa cells [14,15]. While many general outcomes were qualitatively similar, our analysis showed notable cell-type specificity. For example, the degree of regulation mediated by hnRNPL and SRSF1 varied significantly between the cell lines (Figure 1). Along with differences in RBP activity between the cell lines, the regulated exons had minimal overlap across the cell lines (Supplemental Table S6). These observations suggest that distinct gene expression programs in HeLa, HepG2, and K562 cells result in RBP activity and exon selection. Alternatively, the abundance of RBPs in each cell line could alter exon selection. Thus, while an RBP’s ability to interdependently regulate splicing remains constant, cell-type-specific differences in alternative splicing appear to be mediated through altered expression of splicing regulators and target genes.

## Data Availability

The original contributions presented in this study are included in the article/Supplementary Materials. Further inquiries can be directed to the corresponding author.

## Supplementary Materials

The following supporting information can be downloaded at: https://www.mdpi.com/article/10.3390/genes17010078/s1, Figure S1: Total exons regulated in HepG2 and K562 cells; Figure S2: Clustering of splicing factor interdependence preference percentage; Figure S3: Statistical analysis of splicing factor interdependence in HepG2 and K562 cells; Figure S4: Silencing and enhancing activity of splicing factors in HepG2 and K562 cells; Figure S5: Correlations between exon and upstream intron length and the number of splicing factors acting on an exon; Figure S6: Regulation of the alternative splicing of splicing factors by other splicing factors; Table S1: Data and data links used in analysis; Table S2: Inclusion level differences in affected exons in HeLa cells; Table S3: Inclusion level differences in affected exons in HepG2 cells; Table S4: Inclusion level differences in affected exons in K562 cells; Table S5; Summary of interdependent regulation activity and disease association of independent splicing factors; Table S6: Overlap of affected exons across HeLa, HepG2, and K562 cell lines.

## Abbreviations

The following abbreviations are used in this manuscript:

RBP	RNA Binding Protein
AS	Alternative Splicing
ES	Exon Skipping
SR	serine/arginine-rich
hnRNP	heterogeneous nuclear ribonucleoprotein
HeLa Cells	Henrietta Lacks cell line
HepG2 Cells	Hepatocellular carcinoma cell line
K562 Cells	Erythroleukemia cell line
ENCODE	Encyclopedia of DNA Elements
qRT-PCR	Quantitative Reverse Transcription Polymerase Chain Reaction
