# Supplementary material for: Interdependent Regulation of Alternative Splicing by Serine/Arginine-Rich and Heterogeneous Nuclear Ribonucleoprotein Splicing Factors
Source: Genes (Basel). 2026 Jan 9;17(1):78. doi: 10.3390/genes17010078 (PMC12840759; doi:10.3390/genes17010078)
Supplement: Supplementary file 1 [file genes-17-00078-s001.zip › Supplemental_Figures_and_Table_Descriptions.pdf]

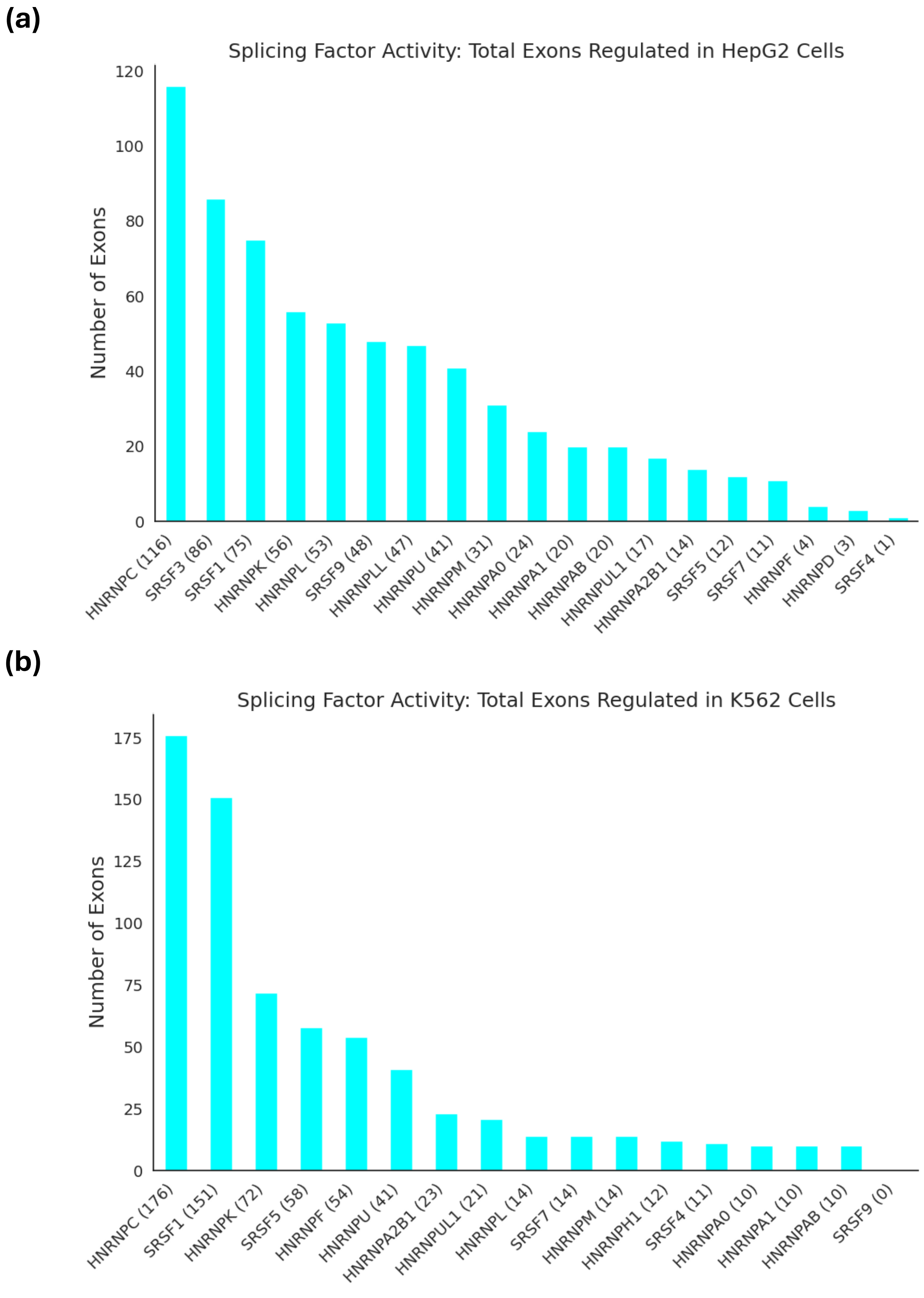

Figure S1. Total exons regulated in HepG2 and K562 cells. **(a)** Bar plot showing the total number of exons with more than 10% change in inclusion level and a FDR ≤ 0.05 upon RBP knockdown in HepG2 cells. **(b)** The same as **(a)** but for K562 cells.

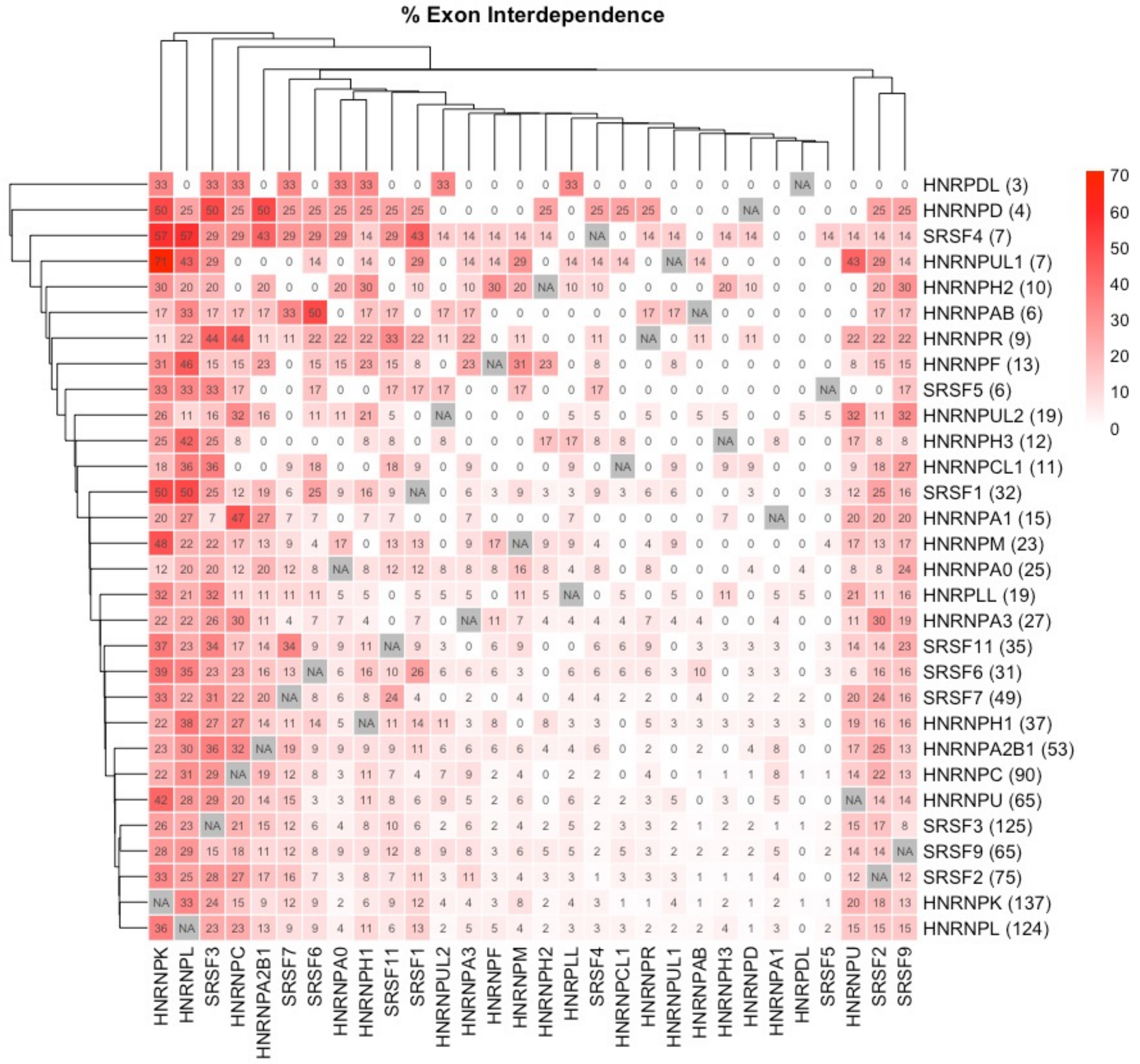

Figure S2. Clustering of splicing factor interdependent regulation preference percentages. Clustering of the interdependence matrix from Fig. 3b.

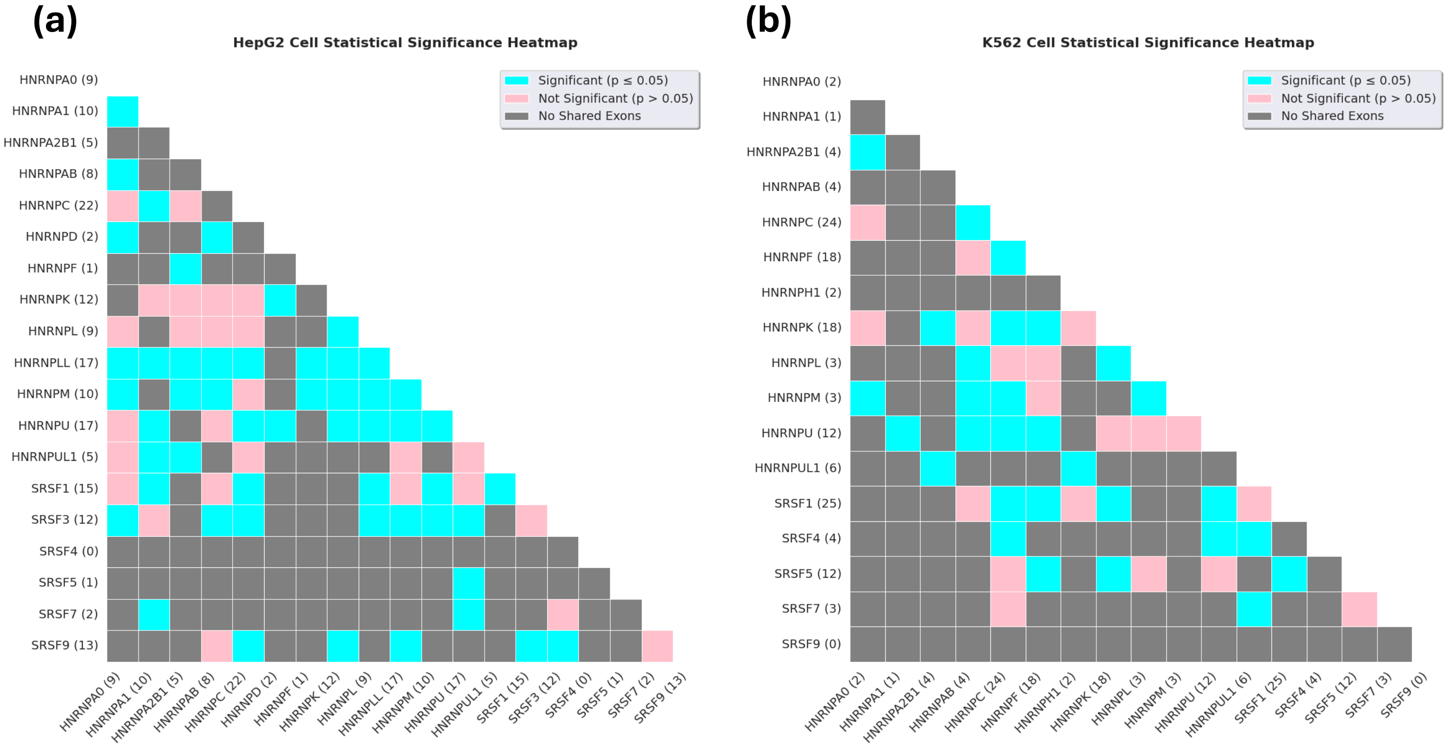

Figure S3. Statistical analysis of splicing factor interdependence in HepG2 and K562 cells. **(a)** Matrix indicating which of the pairs of the 19 splicing factors analyzed show statistically significant interdependent regulation in HeLa cells. The number in parentheses after each RBP name is the total number of exons with AS that are also affected by another RBP. Cyan indicates statistically significant pairs (p <= 0.05), pink indicates statistically insignificant pairs (p > 0.05), and gray indicates no overlap between pairs. **(b)** The same as **(a)** but for the 17 splicing factors analyzed in K562 cells.

(a)

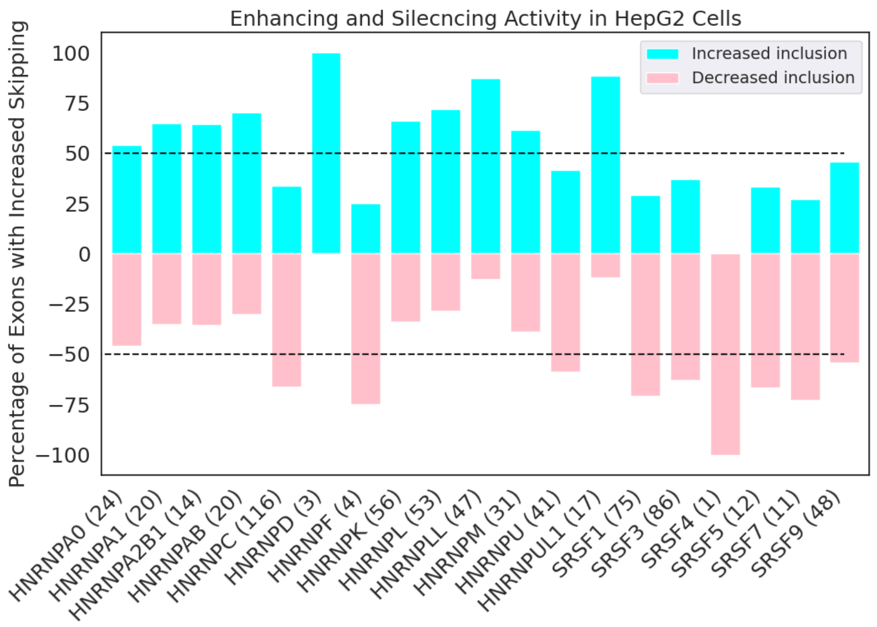

(b)

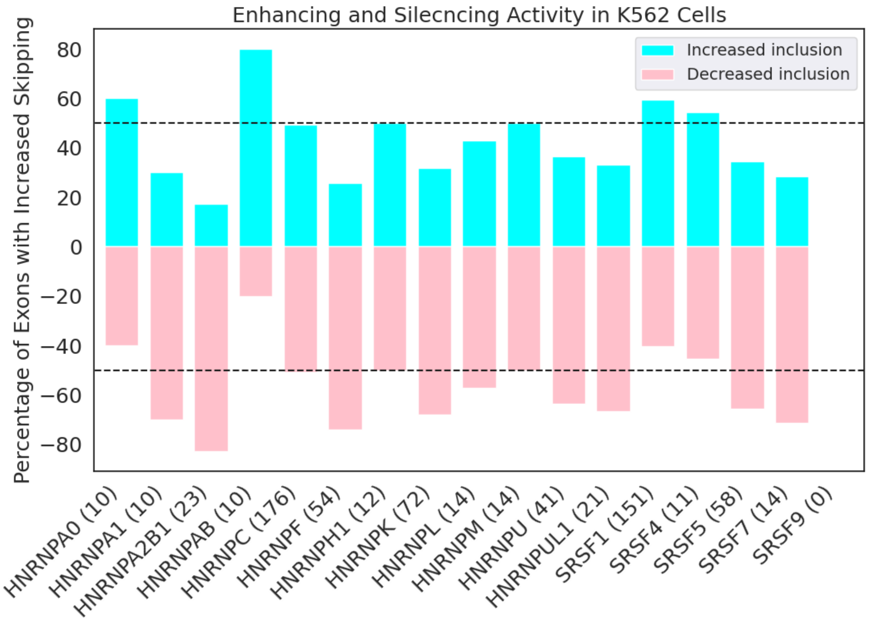

Figure S4. Silencing and enhancing activity of splicing factors in HepG2 and K562 cells. **(a)** Stacked bar graph showing the percentage of affected exons with decreasing (pink) and increasing (blue) inclusion levels upon knockdown of each RBP in HepG2 cells. **(b)** The same as **(a)** but for K562 cells

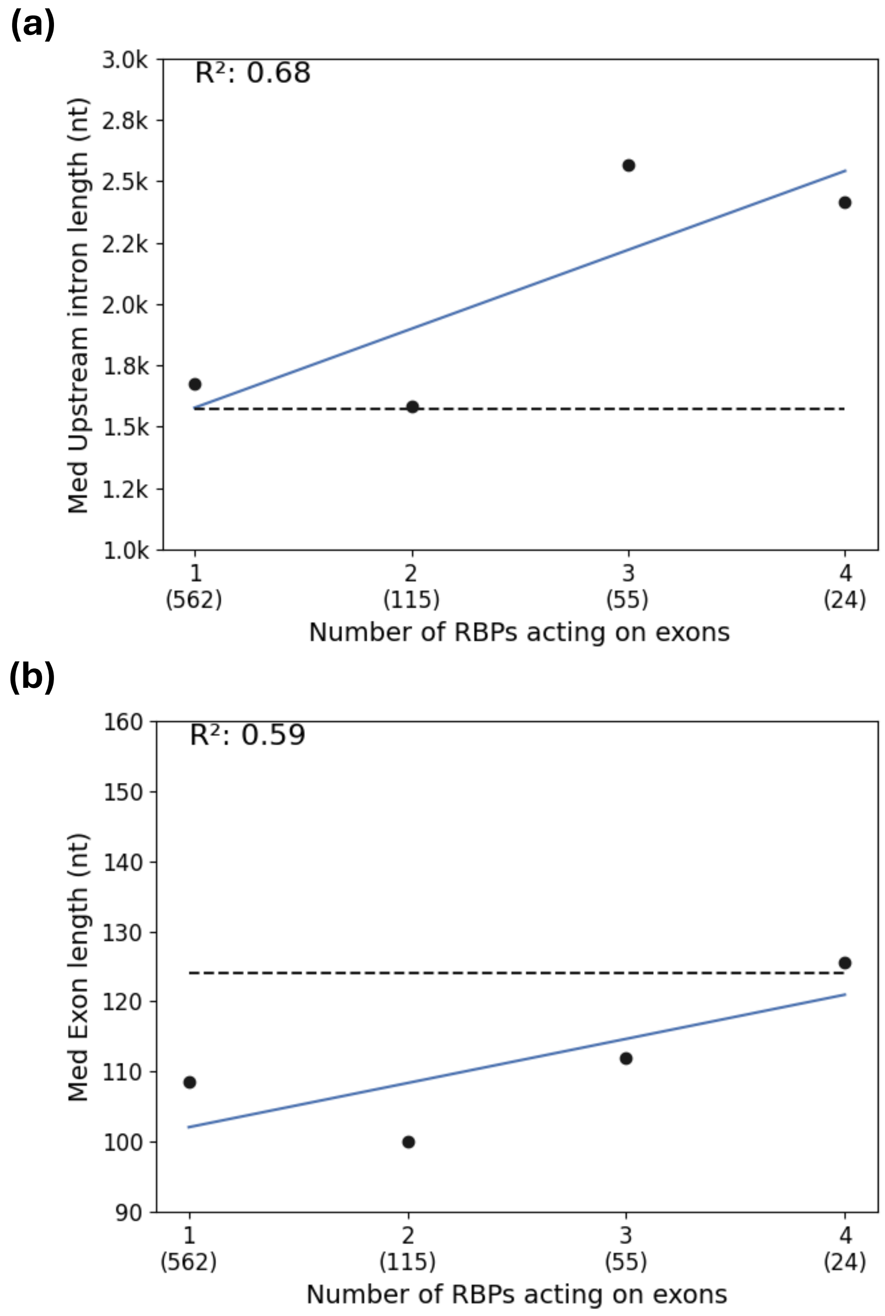

Figure S5. Correlation between exon and upstream intron length and the number of splicing factors acting on an exon. **(a)** Correlation between upstream intron length and the number of RBPs affecting individual exons. The dashed line indicates the median upstream intron length for all internal exons in the human transcriptome. **(b)** The same as **(a)** but for the exon length.

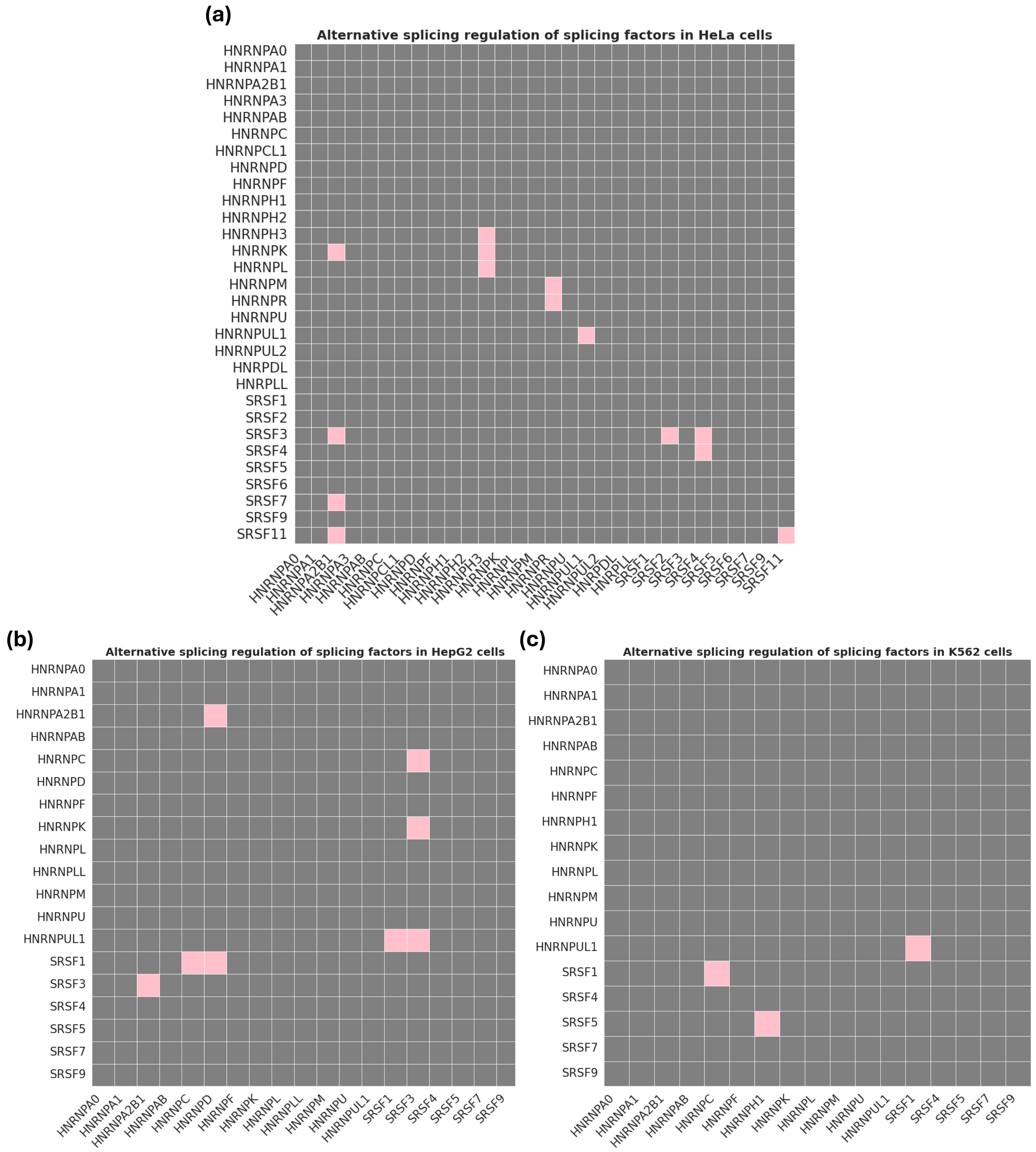

Figure S6. Regulation of the alternative splicing of splicing factors by other splicing factors. **(a)** Heatmap showing which RBPs have changes in exon inclusion upon knockdown of a given RBP in HeLa cells. Pink indicates a change in the splicing of the column protein upon knockdown of the row protein. Gray indicates no change in the splicing of the column protein. **(b)** The same as **(a)** but for HepG2 cells . **(c)** The same as **(a)** and **(b)** but for K5622 cells.

Table S1. Datasets and ascension codes for differential splicing analyses of knockdown data that were used. Sheet 1 contains the full HeLa cell analysis with the inclusion level difference seen by each exon upon the knockdown of a splicing factor. Sheets 2 and 3 contain the links and experimental information for the differential splicing analyses of the splicing factor knockdowns in HepG2 and K562 cells, respectively.

Table S2. Inclusion level differences of affected exons in HeLa cells. Table containing sheets with gene ids, coordinate information, and percent inclusion level difference for all exons with >15% change in inclusion level upon knockdown of the RBP in the sheet name in HeLa cells.

Table S3. Inclusion level differences of affected exons in HepG2 cells. Table containing sheets with gene ids, coordinate information, and percent inclusion level difference for all exons with >10% change in inclusion level and a FDR ≤ 0.05 upon knockdown of the RBP in the sheet name in HepG2 cells.

Table S4. Inclusion level differences of affected exons in K562 cells. Table containing sheets with gene ids, coordinate information, and percent inclusion level difference for all exons with >10% change in inclusion level and a FDR ≤ 0.05 upon knockdown of the RBP in the sheet name in K562 cells.

Table S5. Summary of interdependent regulation for individual splicing factors. Each sheet shows the splicing factors interdependently regulated with each individual splicing factor for the given cell line as well the diseases the splicing factor is known to be associated with.

Table S6. Overlap of affected exons across HeLa, HepG2, and K562 cell lines. Table showing the number of exons that were affected by the knockdown of an RBP in all three cell lines (HeLa, HepG2, and K562) or in more than one of the cell lines.
